# Fiber-Optic Vector-Magnetic-Field Sensor Based on Gold-Clad Bent Multimode Fiber and Magnetic Fluid Materials

**DOI:** 10.3390/ma15207208

**Published:** 2022-10-16

**Authors:** Weinan Liu, Shengli Pu, Zijian Hao, Jia Wang, Yuanyuan Fan, Chencheng Zhang, Jingyue Wang

**Affiliations:** 1 College of Science, University of Shanghai for Science and Technology, Shanghai 200093, China; 2 Shanghai Key Laboratory of Modern Optical System, University of Shanghai for Science and Technology, Shanghai 200093, China

**Keywords:** vector-magnetic-field sensing, surface plasmon resonance, magnetic fluid

## Abstract

A kind of bent multimode fiber (MMF) vector magnetic sensor based on surface plasmon resonance (SPR) was proposed. By plating gold film on the curved part of the bent multimode fiber, the surface plasmon mode (SPM) was excited via a whispering gallery mode (WGM). Fabricating the structure only required bending the fiber and plating it with gold, which perfectly ensured the integrity of the fiber and made it more robust compared with other structures. The sensor used magnetic fluid (MF) as the magnetically sensitive material. Through monitoring the shift of the surface plasmon resonance dip, the as-fabricated sensor not only had a high magnetic field intensity sensitivity of 9749 pm/mT but could also measure the direction of a magnetic field with a high sensitivity of 546.5 pm/°. The additional advantages of the proposed sensor lay in its easy fabrication and good integrity, which make it attractive in the field of vector-magnetic-field sensing.

## 1. Introduction

Magnetic fluid is a kind of stable colloidal material, which is composed of magnetic nanoparticles, surfactant coated on the surface of the particles and carrier liquid. It has the magnetic properties of solid magnetic materials and unique optical properties. Due to its tunable refractive index (RI) characteristics, optical anisotropy and good compatibility with optical fiber, MF has been widely used in optical fiber sensors [1,2,3,4,5]. Recently, many fiber sensors combined with MF have been proposed for magnetic field, temperature and curvature measurement. The employed structures include tapered fibers [6,7,8], Fabry–Perot interferometers [9,10], photonic crystal fibers [11,12], bent fibers [13,14], fiber multimode interferometers [15,16], microfiber couplers [17,18] and Sagnac interferometer [19].

Compared with other structures, the bending structure does not require complex processes of manufacturing (such as mechanical polishing, chemical etching or tapering), which ensures the mechanical integrity of the optical fiber. In addition, it intrinsically meets the prerequisite for vector-magnetic-field measurement, i.e., the optical fiber structure is geometrically non-centrosymmetric [20,21,22]. In addition, the bending structure automatically enables the reflective-type configuration and can be regarded as a sensor probe, which is more pragmatic compared with other structures. Moreover, the small size of the structure and the reflection-like probe are convenient for magnetic field detection in a narrow space.

The bending structure can also easily stimulate whispering gallery modes (WGMs) within the cladding [23,24,25,26], which makes it possible to combine the bending structure with a surface-plasmon-resonance (SPR)-sensing system. It is well known that sensors based on SPR have relatively higher sensitivity [27,28,29,30], which can measure very tiny changes in the environmental RI around the structure. Though many sensors based on bent fiber have been proposed and demonstrated, the SPR effect has not attracted much attention from researchers. Most of the sensing mechanisms of those sensors have been based on Mach–Zehnder interference via coupling between core mode (CM) and WGM [31,32]. As the structures belong to the interference type, there is a free spectral range (FSR), which leads to a limited detection range of the sensor (i.e., the main deficiency of this kind of device). This is due to the very small interval between the two neighboring interference dips [33].

In view of the above-mentioned facts, we proposed and experimentally demonstrated a kind of vector-magnetic-field sensor based on a MF-clad bending fiber-optic structure with SPR, which possessed the benefits of both bent fiber and SPR such as easy fabrication, good integrity and high sensitivity.

## 2. Fabrication and Sensing Principle

**Figure 1:**
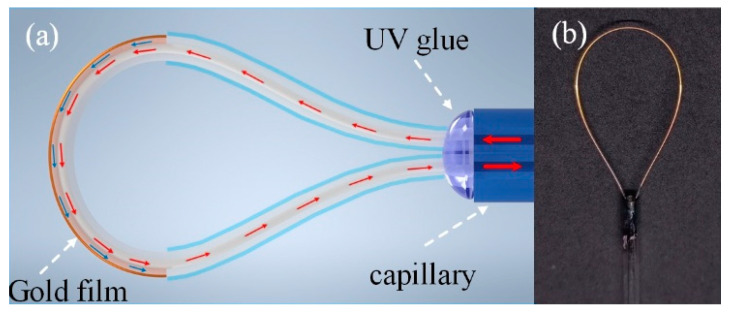
(**a**) Sensing structure of gold-clad bent multimode fiber; (**b**) Photograph of the sensor sample.

The structure of the proposed sensor is shown in Figure 1, which was made of multimode fiber (MMF, SI 105/125-15/250, Yangtze Optical Fibre and Cable Joint Stock Limited Company, Wuhan, China) with cladding and with core diameters of 125 μm and 105 μm, respectively. It has been reported that a too large or too small bending radius is not conducive to producing a clear SPR resonant dip [34]. On the other hand, it is necessary to avoid fiber fracturing caused by bending when the diameter is too small. After a series of pre-experiments, we made three sensors with nominal bending diameters of 8.0 mm, 9.0 mm and 10.0 mm (the actual measured bending diameters were 7.8 mm, 8.9 mm and 10.1 mm, respectively), which are hereafter referred to as sensor 1, sensor 2 and sensor 3, respectively.

To fabricate the sensing structure, the coating of a piece of MMF was stripped to obtain the uncoated fiber with lengths of 12.6 mm, 14.1 mm and 15.8 mm, respectively. Then, the coating-removed fiber was bent and inserted into the capillary of an appropriate length. The fiber at the end of the capillary was stretched to control the bending radius and the position of the fiber. After reaching the appropriate position, UV glue was dropped into the capillary and fixed by UV-irradiation. By this simple method, the coating-stripped fiber was bent into a semicircle and the length of the coating-stripped fiber was equal to the circumference of the semicircle. So, it was convenient and simple to control the bending diameter of the sensor probe. Then, gold film was plated on the surface of the bent MMF using an ion-sputtering apparatus (ETD-900M, Vision Precision Instruments, Beijing, China) with a 10 mA sputtering current for 90 s. The thickness of the deposited gold film was about 17.3 nm. It should be pointed out that the thickness and roughness of gold film will influence the SPR effect and thus the sensing performance. Generally, the sensitivity of the sensor increases with the thickness of the film. However, the normalized depth of the resonance dip decreases at the same time, which is caused by the enhanced evanescent field energy absorption in the gold film [35]. Moreover, the full width at half-maximum of the resonance dip will increase with the gold film thickness, which will reduce the measurement accuracy of the sensor.

**Figure 2:**
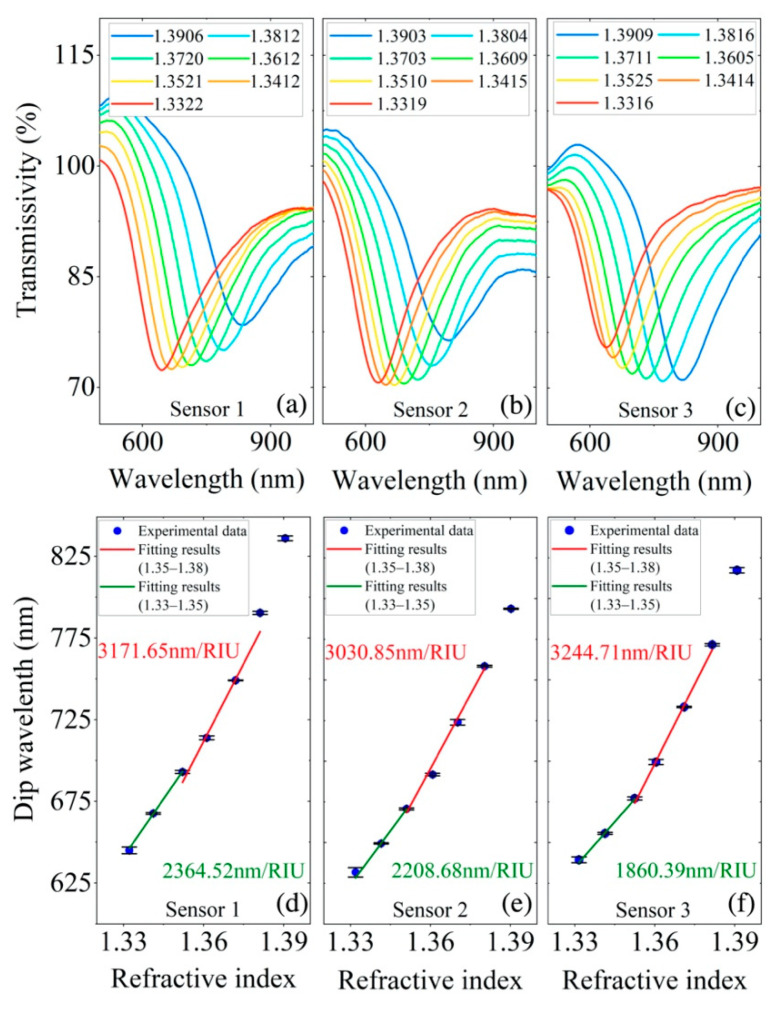
Spectra of the as-fabricated sensing probes clad with liquids of different RIs: (**a**–**c**). Relationship between resonance wavelength and RI: (**d**–**f**). (**a**,**d**), (**b**,**e**) and (**c**,**f**) correspond to sensor 1, sensor 2 and sensor 3, respectively.

Before being packaged in a capsule filled with MF, the RI response of the as-prepared sensors was measured by placing the sensor in liquids with different concentrations. A mixture of glycerol and water with different proportions were used as the liquids with different RIs. Then, the MF with an appropriate RI was chosen, which guaranteed that the magnetic-field-modulated RI range lay within the linear response interval of the SPR dip wavelength. The typical SPR spectra of the as-fabricated three sensors clad with liquids of different RIs (1.33–1.39) are shown in Figure 2a–c. The SPR resonance dip was very obvious. Three tests were performed for each RI to verify their robustness and repeatability. When the RI of the external solution increased, the dip redshifted. In order to facilitate the application of sensing, linear fitting in two limited variation ranges was employed. A very good linearity and a very small error were obtained. The three sensors had high sensitivities in the RI range from 1.35 to 1.38, which were 3171.65 nm/RIU, 3030.85 nm/RIU and 3244.71 nm/RIU, respectively (see Figure 2d–f).

It is obvious from Figure 2a–c that the contrast of SPR dip decreased with the RI, which may be assigned to the increased loss when the ambient RI approached that of the fiber. This also increased the sensitivity in a high RI environment, as shown in Figure 2d–f.

After the RI measurement, the structure was packaged in a capsule filled with MF. Water-based MF with surfactant-coated Fe_3_O_4_ nanoparticles (EMG 605) was employed in this work. Although the larger the RI of the MF is, the better the sensing performance of the structure will be, the resonant dip broadened under a higher magnetic field (i.e., a higher RI), which was counterintuitive. In our experiments, the MF was diluted with deionized water. The achieved RI was about 1.360 (measured by a refractometer A670, Jinan, China), which was within the linear range of the response of the SPR resonance wavelength.

Due to the bending effect, the WGM can be excited within the cladding, and then surface plasmon mode (SPM) can be excited by the evanescent field of the WGM. The coupling between the CM within the core and the SPM within the gold film is mediated by the WGM. So, coupling among the CM, cladding of the WGM and symmetric SPM can be realized simultaneously. Since the effective refractive index (ERI) of SPM depends on the surrounding medium RI (n_s_), the ERI of the WGM-SPM hybrid mode also depends on n_s_. Meanwhile, resonance loss will occur when the CM and WGM-SPM hybrid mode are coupled. This leads to an environment-dependent resonant dip. So, the drifting of the coupling wavelength of CM-WGM-SPM is closely related with n_s_ [34].

The evanescent field of WGM can penetrate the metal film and then excite the SPR if the following phase-matching condition is fulfilled [27]:

(1) 2πλncsin(ϕ)=Re[2πλ(εmns2εm+ns2)12],

where λ represents the wavelength of the incident light, n_c_ is the RI of the fiber cladding, *ϕ* is the incidence angle, ε_m_ is the dielectric constant of the metal film and n_s_ is the RI of the surrounding medium. The sensitivity of an SPR-based sensor is generally defined as [36]

(2) $$S=\frac{d\lambda }{dn_{s}}=\frac{n_{g}^{4}{\left(\omega_{p}\lambda \right)}^{2}n_{s}}{\pi c\left(n_{s}^{2}-n_{g}^{2}\left(1+n_{s}^{2}\right)\right)\sqrt{-\Gamma^{2}+4\omega_{p}^{2}\frac{n_{s}^{2}-n_{g}^{2}}{n_{s}^{2}-n_{g}^{2}\left(1+n_{s}^{2}\right)}}},$$

where *n_g_* is the effective RI of the guided mode propagating in the optical fiber, *Γ* is the electron relaxation rate given by *Γ* = 1/*τ*, *τ* is the electron relaxation time, *ω_p_* is the plasma frequency and *c* is the light velocity.

**Figure 3:**
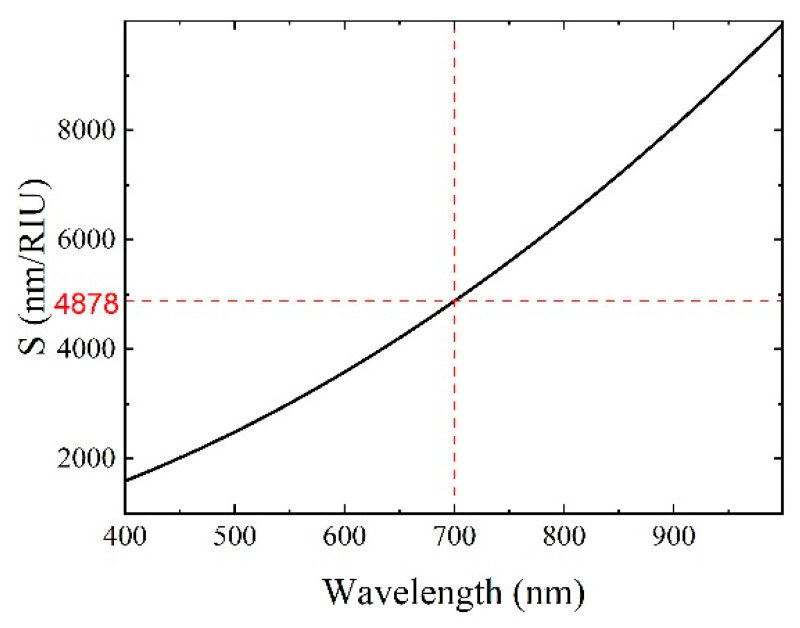
Relationship between theoretical sensitivity S and SPR resonance wavelength.

According to Equation (2), the theoretical sensitivity *S* as a function of λ was calculated and the corresponding results are in plotted Figure 3. The following parameters were taken for the calculation: *ω_p_* = 9 eV/ℏ (ℏ = 6.582119514 × 10^−16^ eV s is the reduced Planck’s constant), *τ* = 14 fs and *n_s_* = 1.360. At the typical resonance wavelength (700 nm), the theoretical sensitivity was 4878 nm/RIU, which was slightly larger than the experimental one.

**Figure 4:**
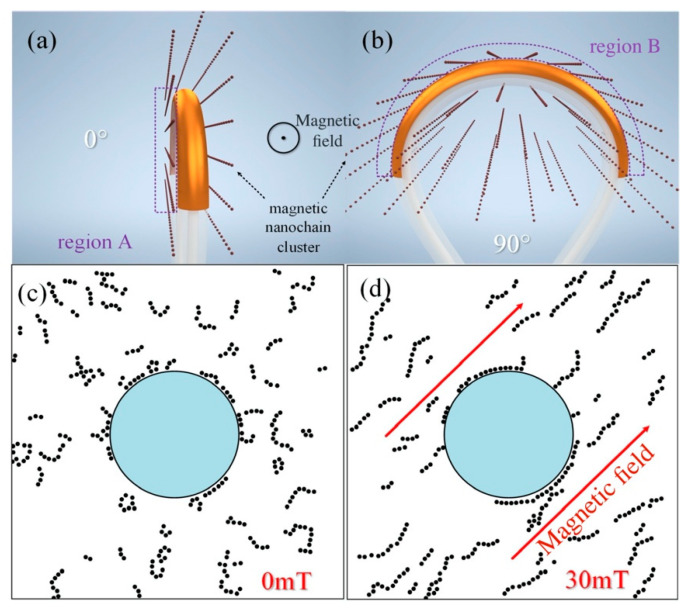
Schematic of nanochain clusters of magnetic nanoparticles under different magnetic field directions: (**a**,**b**); distribution of magnetic nanoparticles around optical fibers simulated with Monte Carlo method: (**c**) H = 0 mT, (**d**) H = 30 mT.

Magnetic nanoparticles in MF will interact with each other and move under the action of a magnetic field. They will form nanochain clusters as is schematically shown in Figure 4, which is closely related with the direction of magnetic field [21,37]. The Monte Carlo method was also used to simulate the distribution of magnetic nanoparticles around the fiber (see Figure 4c,d) [38]. When the magnetic field strength was 0 mT, the magnetic nanoparticles were randomly distributed around the fiber. However, when the magnetic field intensity was 30 mT, nanochain clusters parallel to the magnetic field were formed on both sides of the fiber, where the local RI increased. The RI of the MF around the structure changed with the magnetic field direction as well. In other words, the ERI of the SPM was affected by both the magnetic field strength and direction, which is the basic principle for vector-magnetic-field sensing.

## 3. Experimental Details and Discussion

**Figure 5:**
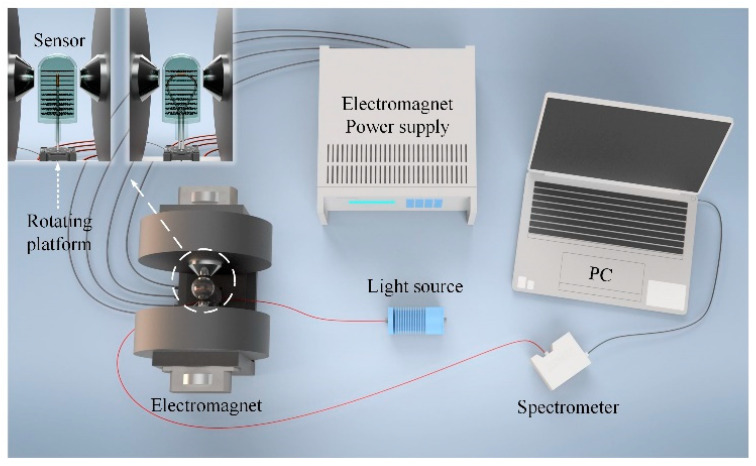
Experimental setup for investigating the sensing properties of the vector-magnetic-field sensor.

Figure 5 shows the experimental setup for investigating the sensing properties of the as-fabricated vector-magnetic-field sensor. The light source was tungsten halogen with a wavelength range from 360 nm to 2400 nm (HL-2000, Ocean Insight, Orlando, FL, USA). The sensor was fixed to a rotating platform placed in the center of the electromagnet. The magnetic field intensity was adjusted by changing the power supply current.

**Figure 6:**
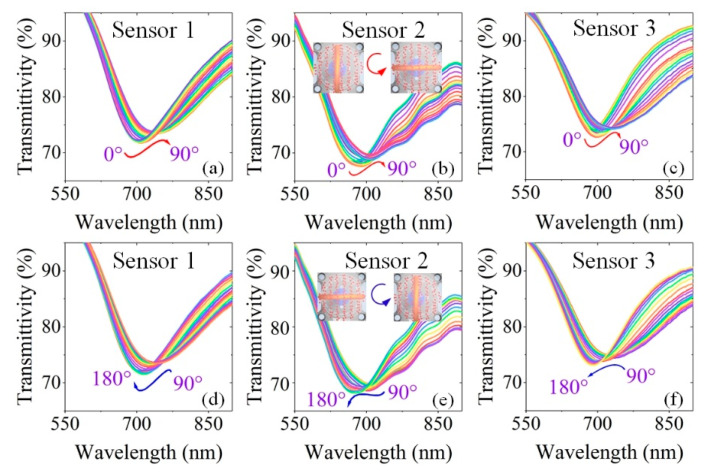
Transmittance spectral response to magnetic field direction under magnetic field intensity of 5 mT for sensor 1 (**a**,**d**), sensor 2 (**b**,**e**) and sensor 3 (**c**,**f**).

**Figure 7:**
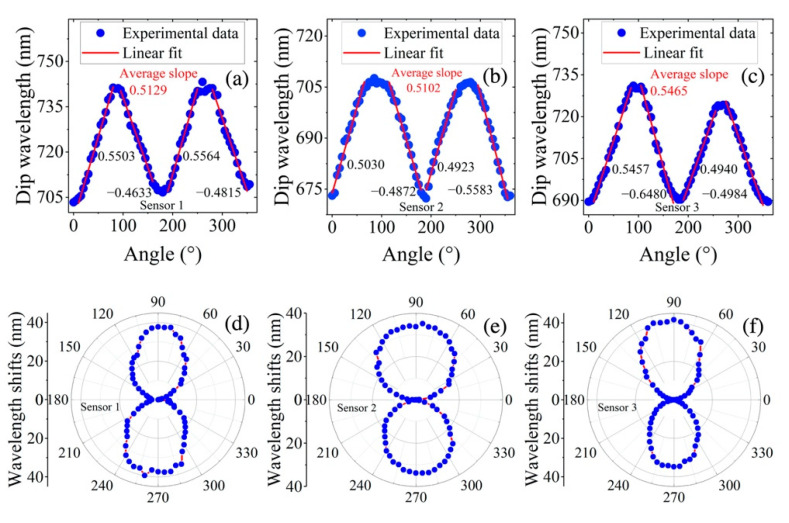
(**a**–**c**) Dip wavelength as a function of magnetic field orientation angle and the linear fitting of experimental data for sensor 1, sensor 2 and sensor 3; (**d**–**f**) shift of dip wavelength with magnetic field direction for sensor 1, sensor 2 and sensor 3 in polar coordinate system.

To verify the vector-magnetic-field-sensing characteristics, the magnetic field intensity was fixed at 5 mT and the magnetic field direction was rotated from 0° to 360° with a step of 5°. The angles 0° and 90° indicated that the plane of the bent-ring was parallel to and perpendicular to the magnetic field direction, respectively (see Figure 4 for the definition of direction). Figure 6 shows the typical transmission spectra of the as-fabricated three sensors at different magnetic field directions. Considering the spectral periodicity of the magnetic field direction ranged from 0° to 360°, Figure 7 only shows the spectra for the magnetic field direction in the range 0°–90° and 90°–180°. When the sensor’s angle (the bent-ring with respect to the magnetic field direction) was rotated from 0° to 90° or 180° to 270°, all dips redshifted. On the contrary, when the included angle was rotated from 90° to 180° or 270° to 360°, all dips blueshifted.

The above phenomenon can be explained by the uneven distribution of the magnetic nanochain clusters around the fiber, as shown in Figure 4. For the bent SPR structure, the SPM propagated along the outside cladding gold film. The ERI of the SPM mainly depends on the surrounding environment around the gold film. So, the CM–WGM–SPM coupling wavelength also shifted with the change in the surrounding environment. When the bending MMF plane was rotated to be parallel to the direction of the external magnetic field (see Figure 4a), the magnetic nanochain clusters were mainly distributed on both sides of the sensor (Region A in Figure 5) and the concentration of MF in the region above the sensor (Region B in Figure 4, i.e., the outside area of the bent part) decreased. Then, the ERI of the SPM decreased synchronically. When the bending MMF plane was rotated to be perpendicular to the direction of the external magnetic field (see Figure 4b), the magnetic nanochain clusters were mainly distributed in the region above the sensor (Region B in Figure 4) and the MF concentration therein increased. With the increase in the MF concentration, the RI increased, which resulted in the increase in the ERI of the SPM. These results were in good agreement with the experimental phenomena and the theoretical prediction from Equation (2).

To be clearer, the variation in the dip wavelength with the magnetic field direction is explicitly depicted in Figure 7. The dip wavelength varied periodically with the magnetic field direction. At 0°, 90°, 180° and 270°, the variation in the dip wavelength with direction was slight, while the variation was very remarkable at around 45°, 135°, 225° and 315°. As the magnetic field direction changed from 0° to 90°, the magnetic nanochain clusters moved from both sides of the sensor (Region A in Figure 4) to the region above the sensor (Region B in Figure 4) and the RI changed significantly, so the directional sensitivity was very high (at around 45° or 225°). For the cases that the magnetic nanochain clusters were mainly distributed in the regions near both the sides or above the sensor, the RI changed slightly, so the directional sensitivity was low (at around 0° or 90°). The sensitivity corresponding to the magnetic field direction in the ranges of 0°~90°, 90°~180°, 180°~270° and 270°~360° was obtained through linear fitting of the experimental data, and the average highest direction sensitivity was 0.5465 nm/° (see Figure 7c). In a polar coordinate system, the response of the dip wavelength shift with respect to the orientation angle exhibited an “8” shape (see Figure 7d–f). The directional response of the three sensors was very similar, but the shape of the wavelength drift with respect to the magnetic field direction for sensor 2 was more standardized (symmetrical) than those for sensor 1 and sensor 3. Thus, sensor 2 could measure the magnetic field direction more accurately. The slight asymmetry for the wavelength shift with respect to the magnetic field direction for sensors 1 and 3 may be assigned to the uniformity of the magnetic field produced by the electromagnet and the slight offset of the sensor position during the rotation, which led to the low mirror symmetry for sensor 1 and sensor 3.

**Figure 8:**
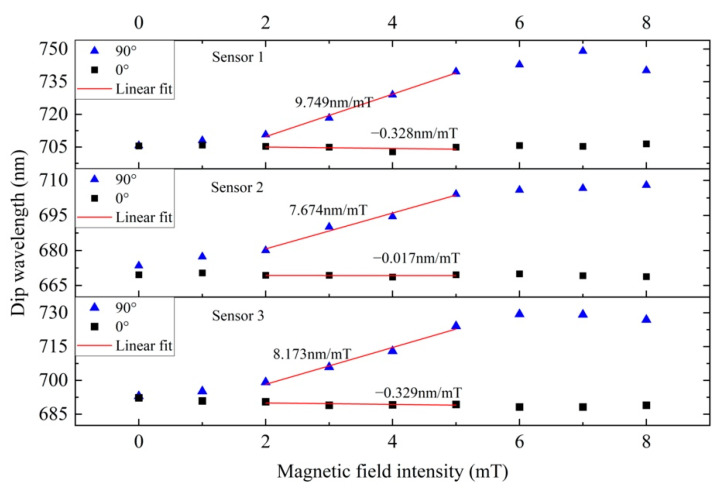
Shift of dip wavelength with magnetic field intensity at 0° and 90°.

Then, the magnetic field direction was fixed at 0° and 90°. The magnetic field intensity increased with a step of 1 mT. The response of the sensors to the magnetic field intensity is characterized in Figure 8. When the magnetic field direction was at 0° and 90°, the dips of sensors redshifted and blueshifted with the increase in the magnetic field intensity, respectively. At 0°, the variation in the dip wavelength with respect to the magnetic field intensity was slight, while the variation was very remarkable at 90°. At the 0° and 180° directions, the bending MMF plane was parallel to the magnetic field direction. As the magnetic nanochain clusters were mainly distributed on both sides of the sensor, the number of magnetic nanoparticles near the gold film at the outside region of the bent fiber was small. Therefore, even if the magnetic field intensity increased, the MF concentration near the gold film changed slightly, as did the ERI of the SPM. Thus, there was only a slight drift in the dip wavelength at this direction. Contrarily, at the 90° and 270° directions, the bending MMF plane was perpendicular to the magnetic field direction. The magnetic nanochain clusters were mainly distributed in the region above the sensor, where the number of magnetic nanoparticles was large. When the magnetic field intensity changed, the MF concentration changed obviously, as did the ERI of the SPM. So, the sensor had a good magnetic field response at 90°. The highest sensitivity was obtained at 9.749 nm/mT at 90° for sensor 1 (see Figure 8). In brief, the experimental results showed that the proposed sensing probe could detect not only the magnetic field intensity but also the magnetic field direction.

For comparison, the sensing structure, fabrication method and sensing performance of various vector-magnetic-field sensors are listed in Table 1. Compared with other structures, the proposed vector-sensing structure in this work had the highest sensitivity and a better mechanical strength without destroying the integrity of the fiber. On the other hand, we also noticed that the detection range of our as-fabricated device was relatively small. Thus, the proposed structure may be more appropriate for applications in fields where high sensitivity is required but the magnetic field varies in a small range, such as biomedicine, medical diagnostics, mineral searches and archaeological excavations [39,40].

Finally, we would like to point out that the ambient temperature was kept constant in this work. However, the RI of the MF is also closely related to temperature. The thermo-optical coefficient of the MF was around −2.4 × 10^−4^ °C^−1^ [41]. So, temperature calibration or compensation may be necessary for applications in a harsh environment and/or high-precision applications. In addition, under a vibrating environment, the magnetic nanoparticle agglomeration will be destroyed to certain extent even though the magnetic field is unchanged. Then, the RI of the MF is affected by the vibration as well. Thus, the sensing performance will be influenced by the vibration. Therefore, anti-vibration design is necessary for the case when a high precision is required.

## 4. Conclusions

In conclusion, a gold-clad bending MMF structure fixed in a vessel filled with MF was used for vector-magnetic-field sensing. Coupling among the CM, WGM and SPM could be realized simultaneously, which resulted in the resonant dip wavelength being closely related to the environmental RI. The magnitude and direction of the magnetic field could be measured by detecting the shift of the dip wavelength. The sensitivity in terms of direction and intensity were 546.5 pm/° and 9749 pm/mT, respectively. The sensor has the advantages of high sensitivity, easy fabrication and good integrity.

## Figures and Tables

**Table 1:** Sensing performance of various optical fiber vector-magnetic-field sensors.

Sensing Structure	Fabrication Method	Detecting Range	Maximum Sensitivity	Reference
STS + lateral-offset	Offset splicing	0–16 mT	222.0 pm/mT	[21]
Tilted fiber grating	UV inscribed	0–15 mT	260 pm/mT	[42]
SMF fused with capillary	Tapered	0–110 mT	112 pm/mT	[43]
SPF-SNS	Side-polished	0–30 mT	2370 pm/mT	[44]
TFBG + gold film	Gold plating	0–18 mT	1800 pm/mT	[37]
FP + lateral-offset	Offset splicing	0–9 mT	4.63 pm/mT	[45]
D-shape fiber + gold film	Side polished + gold plating	0–23 mT	598.7 pm/Oe (5987 pm/mT)	[46]
Wedge-shape + gold film	Tip polished + gold plating	0–22 mT	1100 pm/mT	[47]
Bent MMF + gold film	Bent + gold plating	0–8 mT	**9749 pm/mT**	This work

## Data Availability

The data that support the findings of this study are available from the corresponding author upon reasonable request.
